# The flexibility of Apicomplexa parasites in lipid metabolism

**DOI:** 10.1371/journal.ppat.1010313

**Published:** 2022-03-17

**Authors:** Serena Shunmugam, Christophe-Sébastien Arnold, Sheena Dass, Nicholas J. Katris, Cyrille Y. Botté

**Affiliations:** Apicolipid Team, Institute for Advanced Biosciences, CNRS UMR5309, Université Grenoble Alpes, INSERM U1209, Grenoble, France; Joan and Sanford I. Weill Medical College of Cornell University, UNITED STATES

## Abstract

Apicomplexa are obligate intracellular parasites responsible for major human infectious diseases such as toxoplasmosis and malaria, which pose social and economic burdens around the world. To survive and propagate, these parasites need to acquire a significant number of essential biomolecules from their hosts. Among these biomolecules, lipids are a key metabolite required for parasite membrane biogenesis, signaling events, and energy storage. Parasites can either scavenge lipids from their host or synthesize them de novo in a relict plastid, the apicoplast. During their complex life cycle (sexual/asexual/dormant), Apicomplexa infect a large variety of cells and their metabolic flexibility allows them to adapt to different host environments such as low/high fat content or low/high sugar levels. In this review, we discuss the role of lipids in Apicomplexa parasites and summarize recent findings on the metabolic mechanisms in host nutrient adaptation.

## Introduction

Lipids are one of the most predominant constituents of any living organism and are crucial for cell development and division. They serve as the building blocks of biological membranes, such as those from intracellular organelles. Lipids are also major signaling molecules and serve a central storage moiety for the generation of energy and new membranes. Lipids are therefore essential in the pathogenesis of infectious diseases [1]. Apicomplexa parasites, which are responsible for major social and economic burdens, are no different in this need for substantial amounts of lipids for survival. *Plasmodium* sp. and *Toxoplasma gondii* both belong to this phylum and are the infectious causative agents of malaria and toxoplasmosis, respectively [2,3]. These obligate intracellular parasites have 2 distinct life cycles, either alternating between asexual and sexual life stages, and/or replicative versus low replicative life stages (more particularly for *T*. *gondii*: highly replicative in any nucleated cells from warm-blooded animals versus chronic stages nondividing cells), with distinct organism/cellular requirements, as well as specific replicative and metabolic profiles.

**Fig 1:**
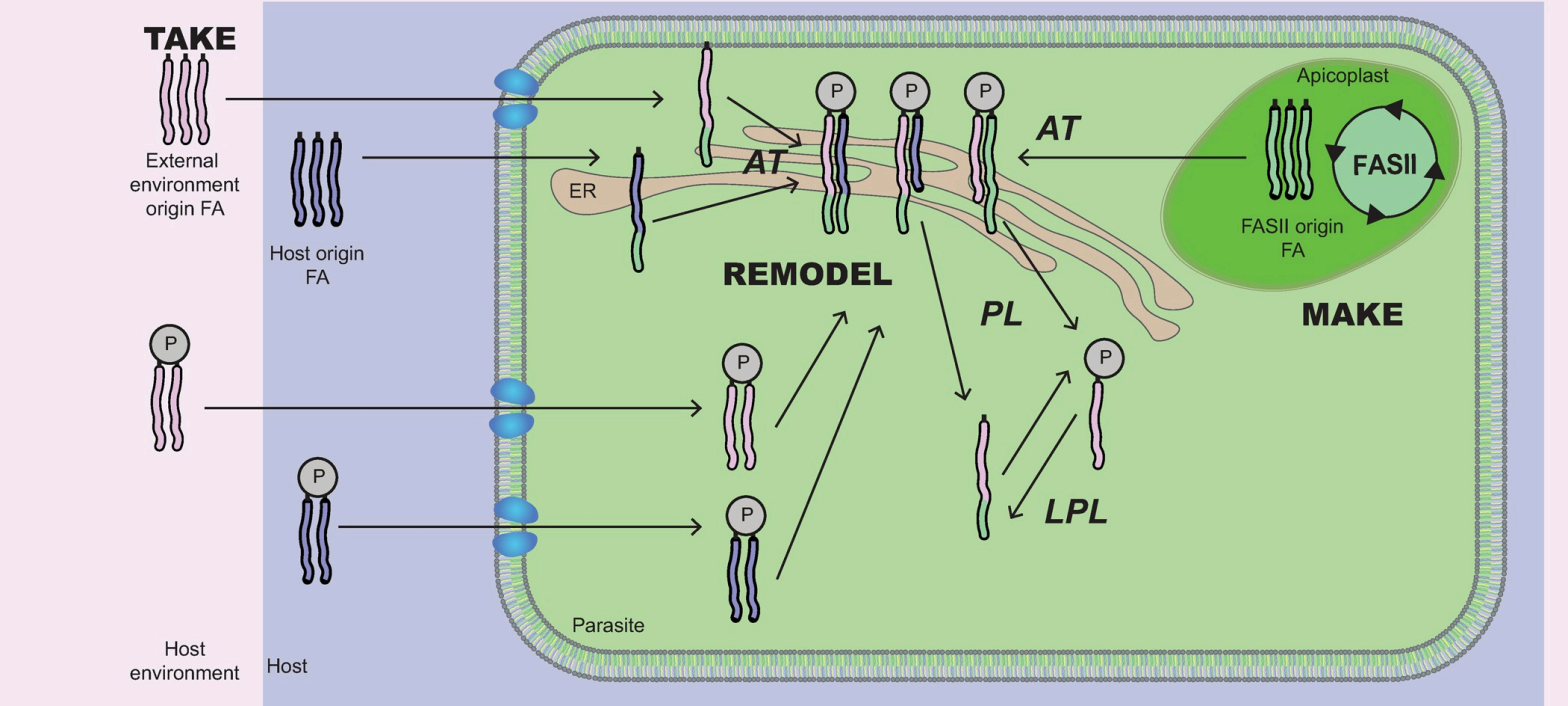
“Make, take, and remodel”; the central dogma explaining how the parasite is able to scavenge lipids from the host environment, the host itself (“Take”) while also synthesize them de novo (“make”). FA elongation and a patchwork assembly mechanism in the ER contribute further to glycerolipid metabolism in the parasite (“remodel”). AT, acyltransferase; FA, fatty acid; LPL, lysophospholipase; PL, phospholipase.

All these life stages are highly dependent on large and specific amounts of lipids, as well as maintaining a specific lipid homeostasis to sustain intracellular development, membrane/organelle biogenesis, and parasite survival and propagation. Parasites also require lipids as signaling molecules, notably to regulate and control the timely release of the secretory organelles called micronemes, which trigger active invasion and egress mechanisms of the parasite in and from their host cell. Importantly, these parasites can metabolically adapt to changing host nutrient environments, setting up a unique metabolic program upon the host nutritional status and, hence, continue to optimally propagate. The unique lipid metabolism of these parasites has been thoroughly characterized by a so-called “make and take” paradigm, where lipid acquisition is met by a combination of de novo–made lipids (“make lipids”) and scavenged lipids from the host (“take lipids”) [4] (Fig 1). Furthermore, recent studies uncovered that the parasites’ survival is governed by their ability to salvage and synthesize simple fatty acyl chains and carry out an obligate “patchwork” assembly with lipids originating from both sources to generate most lipid species and thus maintain the essential parasite lipid homeostasis [5]. Importantly, recent work revealed that the parasite metabolic ability to scavenge, make de novo and reassemble lipids, are regulated, and controlled via sensing and metabolic adaptation of the parasite toward the nutritional status of the host and nutrient availability [6]. Changes in the host nutrient induces a metabolic reprogramming of the parasite where different sets of genes and proteins and not a unique one are responsible for parasite adaptation and survival.

This review will primarily cover recent developments in lipid synthesis/salvage in *Toxoplasma* with reference to other apicomplexan parasite for comparison.

## Lipids are essential in Apicomplexa parasites

Originally, these parasites were thought to solely rely on scavenging from the host for lipids, and not to being able to synthesize their own. Instead cellular investigations, genome mining, and biochemical investigations revealed the presence of a nonphotosynthetic plastid, the apicoplast, harboring a prokaryotic type II fatty acid (FA) synthesis pathway, which is essential during tachyzoite and becomes dispensable during bradyzoite development [7–11]. The pathway in sexual stages (oocyst formation in cats) remains mainly understudied, and its essentiality is unclear. Unlike tachyzoites, we know that *T*. *gondii* oocysts rely on FA breakdown to fuel the tricarboxylic acid (TCA) cycle through the production of acetyl-CoA to generate energy rather than glycolysis [12].

Further numerous analyses allowed for the “make and take” central dogma of lipid metabolism in Apicomplexa parasites to be described and accepted [4]. The FASII pathway, in *Plasmodium* parasites fast replicating blood stage, is not critical for parasite survival in nutritionally rich environments because NADH-dependent enoyl-ACP reductase (FabI) and FabB/F can be deleted. It is vital, however, during the mosquito and liver stages of the parasites in the rodent and human species, respectively [13,14].

**Fig 2:**
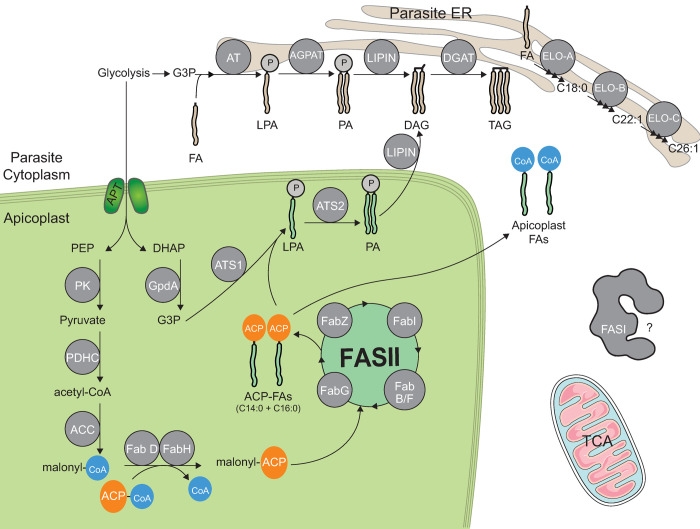
An illustration of the known proteins involved in glycerolipid synthesis in Apicomplexa parasites. PEP and DHAP generated from glycolysis occurring in the cytosol of the parasites enter the apicoplast via the apicoplast phosphate transporter (*Tg*APT1). They are then converted to pyruvate and G3P by a PK and GpdA, respectively. A PDHC (only present in the apicoplast [91]) is responsible for the formation of acetyl-CoA from pyruvate. ACC converts this to malonyl CoA. FabD/H complex leads to the attachment of an ACP and the loss of CoA resulting in ACP-malonyl. The FASII complex (comprised of FabG/B/F/I/Z) are responsible for the formation of short FA chains, mainly C14:0 and C16:0 fatty acyl (FA) ACP-chains. Esterification of FA-ACP from FASII or FA-CoAs from scavenged FAs on a G3P backbone (i.e., acylation) can occur in both the apicoplast by a plant-like acyltransferase *Tg*ATS1 [5], and/or at the ER by a GPAT [93]. It is the first step for the de novo synthesis of all glycerolipids and results in the synthesis of LPA. LPA can be further acylated with another FA chain resulting in PA by ATS2-apicoplast or AGPAT-based ER [6]. LIPIN is responsible for the dephosphorylation of PA into DAG [39]. DAG can be further acylated into TAG by DGATs [94]. Elongation of FA chains can occur in the elongation pathway in the ER via ELO-A/B/C [16]. FASI is present in *T*. *gondii* parasite and not essential during tachyzoite stages and has not been further characterized [15]. ACC, acetyl-CoA carboxylase; ACP, acyl-carrier protein; DAG, diacylglycerol; DGAT, DAG acyltransferase; DHAP, dihydroxyacetone phosphate; FA, fatty acid; GpdA, glyceraldehyde-3-phosphate dehydrogenase; G3P, glycerol-3-phosphate; LPA, lysophosphatidic acid; PA, phosphatidic acid; PDHC, pyruvate dehydrogenase complex; PEP, phosphoenolpyruvate; PK, pyruvate kinase; TAG, triacylglycerol.

Additionally, to the apicoplast FASII, another FA synthesis pathway can be found in *T*. *gondii*, namely the cytosolic eukaryotic type I FA synthase, the FASI. FASI is believed not to be essential in *T*. *gondii* tachyzoite life stages [15] and is completely absent in *Plasmodium* parasites. The contribution of FASI is yet to be fully determined, but it is believed to be inactive during tachyzoite life stage [15] but could play a more important role during the chronic part of the disease, for the development of bradyzoite life stages. Importantly, the parasite depends on an obligate combination of FAs to generate and maintain parasite lipid homeostasis [5]. Indeed, the parasite uses both host-scavenged FAs and de novo–synthesized FA from the relict plastid, the apicoplast, via its prokaryotic FASII pathway [4], to be assembled together and make the bulk of most parasite membrane lipids [5]. FA pools can be further elongated by the addition of 2-carbons units from acetyl-CoA via the ER-based elongases, ELO1, 2, 3 (Fig 2) [16–18]. Glycerolipids synthesis, which leads to the synthesis of phospholipid, the major membrane lipid classes present in the parasite membranes [19–21], is fueled by this essential influx of FAs from the 2 different sources (“make and take”). The synthesis of glycerolipids is composed of an FA “mix and match” process near the ER/cytosol allowing the parasites to maintain a finely tuned balance between the 2 FA sources [5]. This extraordinarily complex and seemingly redundant presence of 2 FA syntheses pathways is in fact not so complex, but instead reflects the parasite’s versatile and diverse capacities to generate and obtain lipids and precursors, crucial for its survival. Indeed, both FA/lipid scavenging and de novo syntheses pathways are essential and allow the parasite to adapt to the adverse or rich nutritional conditions met within the host. Furthermore, such complex pathway of “take, make, remodel, and adapt to the host” involves numerous proteins, which manipulate lipids as part of the general metabolic homeostasis of the parasite within host nutrient changes. Many of these proteins become essential during tachyzoite life stages, while others become involved and pivotal under certain host nutritional conditions, settings lipid acquisition metabolic programs, accordingly, to host nutrient availability.

### Lipids made by the parasite can be influenced by the nutrient environment

Apicomplexa parasites harbor a plant-like plastid acquired from the secondary endosymbiosis of a red algal ancestor [22], the apicoplast (Fig 2). This plastid has lost its photosynthesis ability but kept other essential plant-like metabolic pathways such as the isoprenoid precursor synthesis, i.e., the DOXP pathway, the iron–sulfur cluster synthesis pathway and the prokaryotic type FASII pathway [23]. Bioinformatic reconstruction of the pathway, combined to biochemical analyses, especially using stable isotope ^13^C labeling, in both *P*. *falciparum* and *T*. *gondii*, demonstrated that the parasite imports phosphoenolpyruvate (PEP) acid generated by the parasite glycolysis pathway into the apicoplast using plant-like transporters (APT in *T*. *gondii* and TPTs in *P*. *falciparum*) [5,20,24,25]. PEP is then converted to pyruvate by the pyruvate dehydrogenase complex (PDH, which exclusively exists in the apicoplast and not in the mitochondria [26]) after which converts pyruvate to acetyl-CoA, fueling the FA synthesis pathway and its 4 core proteins (FabB/F, FabG, FabZ, and FabI) allowing the elongation of the growing FA chain [27]. The FASII pathway produces relatively “short” FA chains, mainly C12:0, C14:0, C16:0, and very little C18:0 [5,16]. The identification and characterization of 2 plant-like acyltransferases (so-called ATS1/ApiG3PAT and ATS2) located within the apicoplast revealed the capacity of Apicomplexa parasites to sequentially synthetize then esterify activated FA chains (i.e., Acyl-CoA/ACP) onto a glycerol-3-phosphate backbone resulting in the formation of lysophosphatidic acid (LPA) and, thereafter, phosphatidic acid (PA) [5,6,14,28]. Inducible disruption of *Tg*ATS1 is lethal to tachyzoites and causes a drastic reduction of phospholipid content (approximately 50% reduction) including bulk phospholipid synthesis, leading to the disturbance of most parasite organelles while highly affecting the apicoplast membrane integrity. Nevertheless, *Tg*ATS1 disruption (lethal) versus *Tg*ATS2 disruption (nonlethal) revealed that LPA C14:0 is a major apicoplast product essential for the parasite survival and addition of exogenous LPA C14:0 on ATS1 mutant can rescue the parasite growth compare to LPA C16:0. Similar results were obtained by disrupting the ATS1 of *P*. *falciparum* during liver stage, emphasizing the critical role of the apicoplast lipid synthesis during the important life stage for parasite propagation [28]. In the original chloroplast, acyltransferases are purposed to galactoglycerolipid synthesis essential for chloroplast biogenesis and photosynthetic activity [29] but the lack of such lipids in malaria and toxoplasmosis parasites [19,30] and the repurpose of ATS1/2 [5,6] illustrate the remodeling of the organisms to be conform to an parasitic intracellular lifestyle.

The FA synthesis capacity in the apicoplast is also dependent on host nutrient availability. In *P*. *falciparum*, it was shown that the FASII pathway in the apicoplast is essential during liver and mosquito stage while being dispensable during blood stages [13,31,32]. Contrastingly, transcriptomics analyses in malaria patients showed that under starved conditions, the expression of the apicoplast FASII was highly up-regulated in the human host, suggesting that the pathway could be more versatile than originally thought [33]. It was shown that under minimal lipid environment, which still sustain normal parasite growth, the apicoplast FASII could be metabolically (re)activated and generate medium size FA (C14:0) de novo within the parasite [20,34].

This emphasizes the parasite’s ability to sense host conditions and alter its own metabolic processes to optimize its own development and propagation according to the host environment. In *Toxoplasma*, nutrient availability directly influences the synthetic processes of the apicoplast’s FASII [35–38]. For example, in harsh/low host nutritional conditions, stable isotope labeling of the FASII products showed that FASII is metabolically up-regulated and capable of producing significantly more FAs (C14:0) to compensate for the lack of lipids in the external environment. A similar mechanism was revealed in *P*. *falciparum* blood stages where FASII is reactivated under low host lipid environment, becoming critical for parasite survival in blood stages under these conditions over a long period. Likewise, FASII activity can also be reduced to compensate for elevated levels of FA/lipids scavenged from the host to maintain lipid homeostasis, normal parasite propagation, and avoid death by lipotoxicity [39].

Recent findings have shown that FASII disruption by antibiotics (triclosan; [11]) or gene deletion dehydrogenase complex (PDH), malonyl-CoA-[acyl carrier protein] transacylase (FabD) [40], and FabZ [37] leads to an inhibition of parasite growth, which can be rescued with the supplementation of myristic and palmitic acids, confirmation that these acyl chains are the main end products of the pathway. This was similarly observed in the ATS1 mutant, which is rescued after LPA C14:0 supplementation. This illustrated essentiality of host lipid scavenging and the parasite’s ability to compensate for FASII disruption.

Unlike *Plasmodium* and *Toxoplasma*, *Crytosporidium* behaves atypically where in *it* undergoes sexual and asexual stages in a single host and lacks the apicoplast. Intracellular FA synthesis relies on the giant eukaryotic type I FA synthetase (FASI), which is also conserved in *Toxoplasma* but inactive during rapid tachyzoite life stages and completely absent in *Plasmodium*. Host scavenged “short” FA chains C16:0 are used in *Cp*FASI as a substrate for synthesis of “longer” FA chain up to C22:0 that are then involved in lipid and membrane remodeling. Further, this parasite also possesses typical Apicomplexa elongases, but for elongation up to 2 carbons (not 6) from host-scavenged FAs. The absence of FASII and that the substrate of *Cp*FASI is a host-derived FA, suggests that the parasite in not able to perform synthesis of de novo full-length FA chains and it solely relies on its scavenging capacities [41].

## Fatty acids are also directly scavenged from the host and extracellular environment

Parasite also obtain lipids by scavenging the host cell and its extracellular environment [42–44]. Using radioisotope-labeled lipids or precursors, and fluorescently labeled lipids, it has been shown that scavenged host lipids can be actively imported into the parasite and its organelles including the plasma membrane and the endomembrane system of the parasite [42]. These studies first showed how the parasite can import FA, PLs, and cholesterol. Sphingolipids, which are essential for parasite replication, are another important class of lipids actively scavenged by the parasite from host Golgi-derived vesicles despite the presence of a nonessential sphingomyelin synthase in *Toxoplasma* [45,46]. Furthermore, the cellular and metabolic fate of the scavenged lipids remained unknown. Recent studies showed that *Toxoplasma* can scavenge membrane/lipid material from host lipid droplets hijacked by the parasite [47,48], FA from the host mitochondria [49], and that oleic acid (C18:1) is an important scavenged FA that can kill the parasite by lipotoxicity [50]. Collectively, the existing data were suggesting that the parasite needs to scavenge lipids for survival as confirmed by disruption of the apicoplast FASII and/or complementation by external FA sources [5,6,16,37,40]. The use of metabolic labeling and lipidomic have now shed more light on the nature and the cellular destiny for such imported FA. Amiar and colleagues showed that most major phospholipid classes (PC, PE, and PI) and their molecular species were generated from an obligatory combination of FA directly scavenged from the host. Yet, this was using an indirect method labeling de novo–made FA from the apicoplast, and an actual approach to directly quantify the FA “scavengeome” was missing. Dass and colleagues addressed this issue by directly fueling the host FASI pathway with ^13^C glucose to generate its own ^13^C labeled FA and phospholipids. Using such approaches allowed for monitoring and quantification of the scavenging capacity and deciphered the first parasite scavenged lipidome. This confirmed that very long chain FAs are scavenged [16]. Tachyzoites scavenge considerably more medium and long chain FAs than previously thought, notably unsaturated FAs such as palmitoleic acid (C16:1), oleic acid (C18:1), and paullinic acid (C20:1) [39]. Certain lipids and FAs are not produced by the parasite and must be obtained from the host of their environment [42,44,51]. Most importantly, this approach determined that the parasite constantly scavenges host FAs, which are then stored as triacylglycerol (TAG) in parasite lipid droplets, and then timely mobilized when the parasite most need them during parasite division. Altogether, this allows a constant access to lipid resources without the parasite being affected by potential lipotoxic death. However, using radioisotope and fluorescently labeled lipids, it has also been shown that, beyond being directed to lipid droplets, recruited host lipids can be directed also enter into the endomembrane system of the parasite [52].

Expectingly, the host nutrient environment strongly governs the scavenging capacity of *Toxoplasma* tachyzoites as well as the de novo lipid synthesis capacity. Amiar and colleagues have further shown that under host starvation, the parasites can stimulate “overscavenging,” inducing the formation of giant multivesicular bodies made from deep modifications of host organelles, further directed toward the parasite vacuole and which content is imported in the PV [6]. The identity and composition of giant multivesicular bodies (gMVBs) are still unknown at this stage. However, electron microscopy strongly suggest that they originate mainly from the host nuclear envelope/ER and may somehow allow the parasite to increase its lipid scavenging capacity in the absence of nutrient-rich serum. Further research is required to fully characterize their role in host–parasite interactions specific to the host-nutrient environment.

Mi-ichi and colleagues reported that palmitic acid (C16:0) and oleic acid (C18:1) is the sole combination of FAs for intraerythrocytic asexual proliferation of *P*. *falciparum* parasites [34,53]. These FAs can be further activated to acyl-CoAs by 4 acyl-CoA synthetase (ACS) found in *Plasmodium* sp. genome. Intriguingly, *P*. *falciparum* harbor an additional 9 ACSs, which happen to be encoded in the subtelomeric regions where a high level of recombination occurs such as the *var* genes [54]. This emphasizes their importance for the survival of these life stages [27,54]. Of these ACSs, 2 of them have an apicoplast targeting sequence, but their localization and role in FA synthesis remain unclear. Recently, ACS11 as shown to be critical for parasites survival and been proposed as an interesting drug target, with existing MMV compounds [55].

Exogenous FAs, phospholipids, and those released by phospholipases (PLA2) can be recycled and used in phospholipid and neutral lipid assembly [56–58]. Recent work by 2 independent studies have also shown that *Pf*PLA2 is essential for gametocytogenesis during blood stages [57] and the fertilization process during mosquito stages [59]. Collectively, these studies emphasize that the synthesis of endogenous FAs is not essential for parasite growth and may in fact solely rely on exogenous FAs in blood stage parasites.

### Other classes of lipids also need to be directly scavenged from the host and host environment

**Fig 3:**
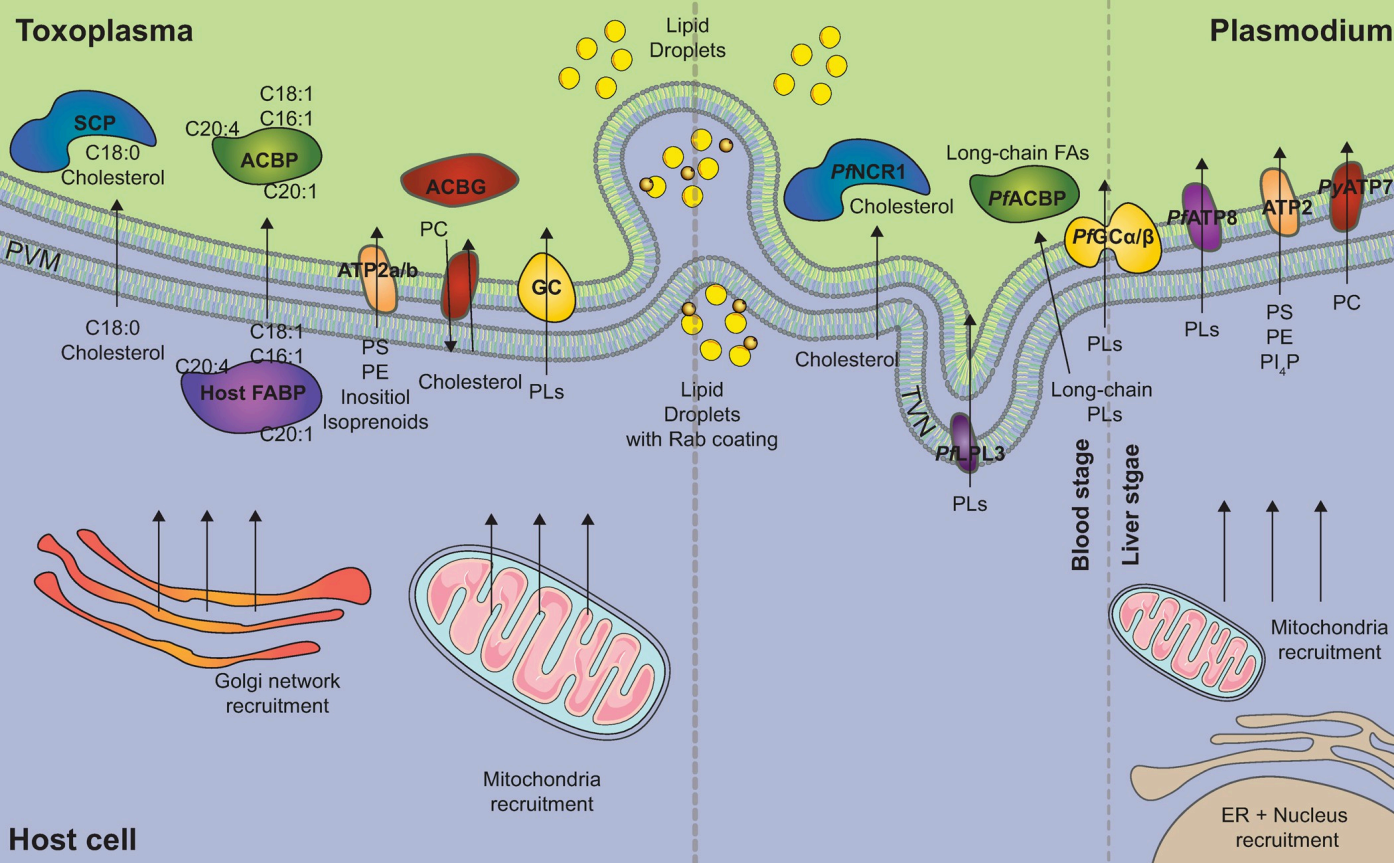
**Scavenging mechanisms known in *T*. *gondii* (left) and *Plasmodium* sp. (right) parasites.** *Toxoplasma* (left): *T*. *gondii* infection displays active recruitment of the host Golgi network and mitochondria to the parasite and is important in nutrient scavenging. Host lipid droplets can be endocytosed into the parasite through the PVM due to their Rab7 coating. Rab vesicles also facilitate the formation of the intravacuolar network found in the PVM. Stearic acid (C18:0) and cholesterol are taken up from the host via an SCP and host FABPs and parasite ACBPs modulate the importation of acyl chains into the parasite and to specific organelles. ABCGs are responsible for the influx/efflux of PC and cholesterol between the host and the parasite. Other lipid transporters are responsible for the scavenging of PS, PE (*Tg*P4-ATPase1-3), choline, sphingolipids, inositol, and isoprenoids; all involved in lipid metabolism. GC is an atypical flippase whose exact mechanism is not yet known but is proposed to be a PL transporter. *Plasmodium* (right): Lipid droplet scavenging occurs in a similar manner to that in *Toxoplasma* parasites. Malarial infection displays active recruitment of the host ER/nucleus and mitochondria to the parasite (not in blood stages). It is suspected that *Pf*NCR1 is important in cholesterol transport from the host. *Pf*LPL3 localized to the intravacuolar TVN. *Pf*LPL3 allows to hydrolyze host phospholipids to generate FA then used for DAG/TAG formation 3 *Pf*ACBPs have been identified that show an affinity to transporting phosphorylated long-chain fatty acyl chains (PLs only). A GC protein also exists in Plasmodium **(***Pf*GCα/β) coupled to a P4 ATPase. ATP8 (in *P*. *falciparum*) and ATP2 (In *P*. *falciparum/chabaudi/berghei*) responsible for the scavenging of an assortment of PLs. *Py*ATP2 is responsible for PC transport into the parasite. ABCG, ATP-binding cassette G family transporter; ACBP, acyl-CoA binding protein; DAG, diacylglycerol; FA, fatty acid; FABP, fatty acyl binding protein; GC, guanylate cyclase; PC, phosphatidylcholine; PE, phosphatidylethanolamine; *Pf*NCR1, *Pf*Niemann–Pick Type C1-Related protein; PL, phospholipid; PS; phosphatidylserine; PVM, parasitophorous vacuole membrane; SCP, sterol carrier protein; TAG, triacylglycerol; TVN, tubulovesicular network.

*T*. *gondii* and *Plasmodium* parasites are auxotroph for cholesterol and must be scavenged from the host or their environment [60,61]. Nishikawa and colleagues identified an acyl-CoA: cholesterol acyltransferase localized to the *T*. *gondii* parasite ER that produces sterol esters and forms lipid bodies (Fig 3). Upon depletion of this protein, free FAs’ levels increased. They were also able to determine that in a host lipoprotein-free environment, there was a decrease in lipid droplet numbers of the parasite. This indicates that the host lipid availability governs neutral lipid synthesis in *Toxoplasma* [62]. Recently, *T*. *gondii* serine hydrolases with esterase and thioesterase activities have been identified and could be acting upon lipids to release FA chains. This also highlights the potential for targeting parasite hydrolases against lipids for therapeutic applications against the parasite [63].

The process by which *Plasmodium* parasites take up cholesterol is still largely unknown. Malaria parasites have moderate need of sterols for optimal development in liver stages, but they can adapt to survive in cholesterol-restrictive conditions by exploitation of accessible sterols derived from alternative sources in hepatocytes to maintain proper infectivity [64]. Recently, a *Pf*Niemann–Pick Type C1-Related protein (*Pf*NCR1) was suggested as an essential molecule for cholesterol transfer intraerytocytic stage parasites [65]. Tomographic imaging results suggest that *P*. *falciparum* initially takes up external cholesterol and/or membrane cholesterol from the inner erythrocyte membrane, after which budding lipid membranes are elongated, migrate to the cytosol, and may fuse with and/or pass through the PVM to eventually reach the parasite body [66].

## Lipid scavenging mechanisms

Identification of proteins and pathways involved in the scavenging of host lipids remain scarce due to the highly divergent and poorly conserved nature of such effectors in *Toxoplasma* and *Plasmodium* parasites. The parasite is able to manipulate the hosts microtubules (Fig 3) to redirect Rab vesicles from the host Golgi network toward the PV [67]. Together with the action of GRA proteins (GRA2, GRA6 …), they then generate an extensive tubular network called the intravacuolar network (IVN) in the parasite parasitophorous vacuole, which contributes to host-lipid scavenging and is dependent from parasite-dense granule proteins [52]. Host lipid droplets and vesicles can also be endocytosed through this parasite-made network due to their Rab7 coating, which is rediverted by the parasite. *Plasmodium* asexual blood stages are also capable for generating trafficking network within the host cell, via extensions of the PVM into the red blood cell cytosol, also known as the tubulovesicular network (TVN), which can promote nutrient exchange including lipids. Indeed, a recent study showed that the parasite expresses a lysophospholipase (LPL) (i.e., lipid-hydrolyzing enzyme) that locates at the TVN to generate FA used for timely membrane biogenesis during schizogony [68]. It has also been shown that the loss of a phospholipase can inhibit the parasites ability to scavenge lipids and further suggests that host lipids are immediately recycled at the point parasite entry at host–parasite interface [47].

The host cell mitochondria and Golgi network have shown to be actively recruited/move toward the PVM right after infection and can contribute to lipid homeostasis [49,61]. Host mitochondria are recruited in close vicinity to the parasite parasitophorous vacuole during *Toxoplasma* infection and limit the parasites’ ability to uptake FA, but it is also suggested that host mitochondria are involved in innate immunity against parasite infection [49]. In host cells lacking mitochondrial fusion, there is an increase in FA uptake [49], hence also pointing that beyond limiting FA supply, mitochondria is also an active source of FA for the parasite. The PVM’s highly porous nature, which allows molecules up to 1.9 kDa into the parasite from the host cytoplasm, is accredited to the secreted parasite proteins GRA17 and GRA23 [69,70]. The parasite can mediate these processes of lipid uptake involving many different host–pathogen interactions through tubular networks and escort proteins. A sterol carrier protein (SCP) called *Tg*HAD-2SCP2 is able to transport cholesterol and oleic acid to be incorporated into neutral lipids [71]. *Toxoplasma* also has 6 ATP-binding cassette G family transporter (ABCG), which localizes differentially within the parasite and are shown to be involved in phospholipid and cholesterol import/efflux [72]. Not only can these parasites scavenge FA, but they can also recruit whole phospholipids [42,73] and neutral lipid stores directly from the host [47]. Phospholipid synthesis is facilitated by the scavenging of precursors such as serine, ethanolamine, and choline from the host environment. These polar headgroups are used by the parasite for the production of phosphatidylserine (PS), phosphatidylethanolamine (PE), and phosphatidylcholine (PC) [55,74]. Chen and colleagues have shown that PE and PS are able to be taken up directly from the host environment, unlike PC that thus has to be generated directly by the parasite from host-scavenged and or de novo–made precursors [75]. A very recent study suggests that parasite lysophopholipase could be used to generate the phosphocholine for parasite PC synthesis from existing lysolipids from the host during *P*. *falciparum* asexual blood stages [68].

PLs and FA are amphipathic molecules, meaning they can disrupt the membrane as they are recruited from the host to the parasite. This dilemma is overcome by carrier proteins such as acyl-CoA binding proteins (ACBPs) and FA binding proteins (FABPs) are such protein classes, which bind to fatty acyl esters and transport them across various cellular compartments [76]. The genome of *Toxoplasma* has no FABP but encodes for 2 ACBPs and are involved in lipid signaling and metabolism. In the absence of the cytosol *Tg*ACBP1, there is a decreased abundance of C18:1, which is predominantly scavenged from the host [73]. The mitochondrial *Tg*ACBP2 plays a key role in cardiolipin metabolism and is critical of the growth and virulence of type II parasites [77]. Both proteins under starvation showed no deleterious effect on tachyzoite growth, indicating it is unlikely they are involved in nutrient adaptation. ATP-dependent PL transporters for PS and PE are present in the plasma membrane of tachyzoites [75].

The *P*. *falciparum* genome annotation revealed the existence of 3 putative ACBPs [78]. Kumar and colleagues show that *Pf*ACBPs have a higher binding affinity to long-chain fatty acyl-CoAs than short chains. They also show that they can bind to phosphorylated lipids but not to the unphosphorylated lipid esters, such as DAG and TAG, suggesting phospholipid transport rather than that of FAs/neutral lipids. Their roles in intracellular transport or host scavenging is still not fully elucidated [79].

To cross membranes in an efficient manner, lipids can be channeled by transporters such as flippases. In *P*. *falciparum* parasites, there are 5 flippases whose role and putative redundancy are currently fragmented [80,81]. *Plasmodium chabaudi* ATP2 has been identified as an essential lipid flippase responsible for PS and PE transport [82]. Homologs in other *Plasmodium* sp. exist but without confirmation of function. Recently, the *Plasmodium yoelii* ATP7 ATPase has been characterized as a phospholipid transporter responsible for the uptake of PC across the plasma membrane in mosquito stage ookinetes [83]. Both *Toxoplasma* and *Plasmodium* sp. possess atypical flippases with dual domains, named GC (*Tg*GC and *Pf*GCα + *Pf*GCβ) consisting of a guanylate cyclase (GC) domain coupled to a P-4 ATPase (flippases). Both are involved in the processes of parasite invasion, egress, and microneme secretion through cGMP and lipid signaling. Briefly, both enzymes can generate cGMP, which activates PKG and a signaling cascade, which includes calcium release and activation of calcium-dependent proteins kinases (CDPKs). The flippase domain of GCs are known to be essential; however, their exact mechanism in the above mentioned process is unclear [55,84,85]. In a study that monitored the in vitro virulence traits of a lab-adapted *T*. *gondii* strain, the early fixation of a P4 flippase gene SNP supported a critical role of lipid homeostasis [35]. Other flippases have been identified in *Toxoplasma*, but their roles have not been fully elucidated [75].

In general, *Toxoplasma* tachyzoite-infected cells are biologically flexible at many levels (genome, epigenetic, transcriptional, protein, and metabolic) to increase their ability to acquire nutrients from their environment and provide them to the parasite. As a good example at the lipid level, once within a cell, tachyzoites increase host synthesis of neutral lipids (TAG) and host lipid droplet numbers [48,49]. This lipid “hijacking” is facilitated via changes at the transcriptional levels of the host, such as the up-regulation of key host enzymes involved in TAG production like acylglycerol-3-phosphate acyltransferase (AGPAT2), acyl-CoA: diacylglycerol acyltransferase (DGAT2), and FA binding transport proteins like FABP3 and FABP5. Similarly, under starvation, parasites exhibit an increase in lipid storage abundance [6]. The low nutritional condition is sensed by the parasite, which attempts to compensate by the up-regulation of FASII lipid synthesis using glucose. The effector *Tg*ASP5, a Golgi-resident aspartyl protease, has been shown to play a role during FBS starvation [6].

Neutral lipids can be acquired from the host through endocytosis via the *T*. *gondii* parasite PVM IVN had Rab7 coating onto their membranes. It is also proposed that probable interaction between Rab effectors and proteins present on PVM in order to promote this event of scavenging [47]. A separate study also should show that LPA accumulates in vesicles that are trafficked through the parasites secretory pathway, suggesting that there is convergence between lipid uptake and the endocytic pathway [86]. The ATS2 (protein involved in LPA to PA conversion) KO mutant has also shown evidence of aberrant endocytosis at the plasma membrane; however, it is presently unclear which, if any, nutrients are taken up by endocytosis during intracellular development [6].

Globally, *Plasmodium* parasites are believed not to take up lipids by typical endocytosis pathways, but characterization of *Pf*EHD and *Pf*LPL1 could suggest that, like *T*. *gondii* parasites, neutral lipids are acquired via endocytosis [87,88]. During *P*. *berghei* liver stage development, there is host cell mitochondria recruitment at the PVM, like in *Toxoplasma*, but in this context to mediate lipoic acid scavenging, which is required for parasite growth [89].

### The balance between membrane biogenesis, lipid storage, and lipotoxicity in nutrient adaptation

**Fig 4:**
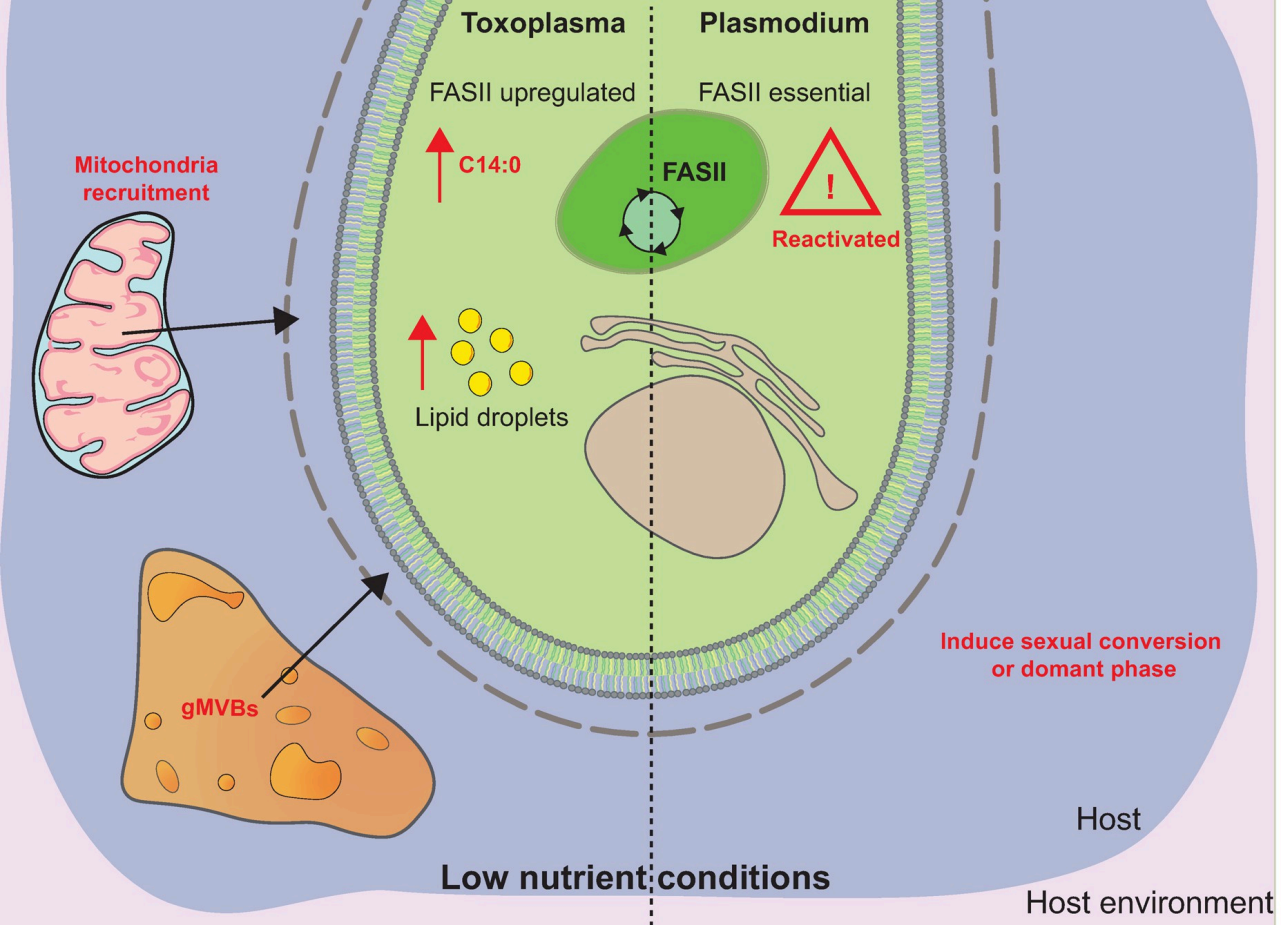
**Cellular changes in parasite (*Toxoplasma*: left; *Plasmodium* sp.: right) in low nutrient conditions.** Mitochondria recruitment and gMVBs are apparent and exacerbated in low nutrient conditions of *Toxoplasma* tachyzoites. The metabolic up-regulation of FASII (resulting in the increase production of C14:0), along with an increase in parasite lipid droplets, is characteristic of low nutritional conditions in the host. In similar conditions, when nutrients are limited in the host environment, *Plasmodium falciparum* asexual blood stages can up-regulate the metabolic activity of the apicoplast FASII, rendering it essential in these conditions. The lack of LPC in the host environment leads to sexual conversion of the parasite. Very little is known about the adaptation of this parasite to changing lipid statuses. gMVB, giant multivesicular body; LPC, lysophosphatidylcholine.

Lipid storage is essential in maintaining this lipid homeostasis within the parasite and is used for the prevention of lipotoxicity or as an efficient energy source in suboptimal growth/culture conditions (Fig 4). The latter can be achieved through the process of β-oxidation in which lipids, in the form of FA, imported into the mitochondria or the peroxisomes, and then broken down into acetyl-CoA. These pool of acetyl-CoAs can then enter the mitochondrial TCA cycle and leads to energy production. The presence of β-oxidation is highly debated in the Apicomplexa field, and many questions [90,91] still exist about the fate of DAG/TAG and the dependence of this process on the host and its proteins/nutrient condition (NB: It is believed that *Toxoplasma* possess a complete beta oxidation pathway in its mitochondrion but lacks the canonical transporter allowing to initiate beta-oxidation, the Carnitine palmitoyl transferase CPT1; the situation is more complex in *P*. *falciparum* where not only CPT1 is missing but also some other members of the beta-oxidation pathway).

A recent study by Primo and colleagues has demonstrated that laboratory adaptation of *T*. *gondii* GT1 strain results in augmented phenotypes driven by selection pressures in the nutrient content of the extracellular environment. They also identified medium chain FAs to be the limiting factor in the extracellular environment, which provided insight into the contribution of FAs in parasite virulence. Lab adaptation evolution experiments performed in 1% FBS, as opposed to 10% FBS showed an up-regulation of genes involved in FA metabolism, specifically those in the FASII pathway. This reiterates the importance of these genes in parasite survival in harsh conditions [35].

The physiological fluctuations of the host nutrient environment are modulated between the equilibrium of both FASII and host-scavenged lipids. Lipids taken up from the host and made de novo by the parasite are reshuffled and remodeled in many different compartments (ER/cytosol/vesicles, etc.) of the parasites by a variety of proteins. Amiar and colleagues demonstrated that disrupting LPA production through its plant-like acyltransferase in the apicoplast leads to a severe impairment of the PL bulk content and composition that leads to a drastic reduction of the abundance of both PC (major membrane component) and PI by at least 50%. To reveal the origin of the PL FA chains, stable isotope labeling of was used in combination with LC-MS based lipidomic analyses (labeled = apicoplast; unlabeled = host). It was revealed that 70% of FA chains (mainly short chains) from both PC and PI are from the apicoplast, while the remaining 30% are from the host. The importance of this ratio of make/take lipids is important to grasp as it is severely altered depending on the nutrient availability and strongly indicates the membrane remodeling ability of Apicomplexa parasites [4,16,21,36,92].

To allow for the remodeling of these lipids, Apicomplexa parasites possess a so-called eukaryotic glycerol-3-phosphate acyltransferase (GPAT; Fig 2), which has been located in the ER (*Plasmodium*) able to assemble LPA [14,93]. This GPAT seems capable of generating LPA with a high specificity for C16:0 and C16:1, both of which originating either from de novo synthesis or being scavenged from the host. In *Toxoplasma*, a putative GPAT and 1-acylglycerol-3-phosphate acyltransferase (AGPAT) locating the parasite ER [6] have been identified but not characterized yet, suggesting that the parasite could be able to reassemble host and de novo lipids in an “eukaryotic” manner to fuel membrane biogenesis. The eukaryotic-type AGPAT was also found in the *Plasmodium* ER, and both the *Toxoplasma* and *Plasmodium* homologs seem essential for the parasite survival [6,14]. On another hand, parasites are also able to massively reroute host-scavenged lipids from membrane to storage to maintain parasite survival through active division in a timely regulation and to prevent parasite death by lipotoxicity. Indeed, recent findings showed that a PA phosphatase in *T*. *gondii* (*Tg*LIPIN) can act as a “switch” and a “metabolic scale” by converting PA to diacylglycerol (DAG), ultimately sustaining bulk TAG synthesis by fueling DAG to diacylglycerol acyltransferase (DGAT) affecting formation of parasite lipid droplets [39,94]. DAG can also be converted to cytidine diphosphate-diacylglycerol (CDP-DAG, pools that are present in the apicoplast or the ER), which serves as a major precursor for phospholipid synthesis [95]. In a low lipid environment, the deletion of *Tg*LIPIN in the parasite leads to a decrease in lipid droplets and TAG levels, ultimately leafing to gross membrane malformations and parasite death by lipotoxicity. Intriguingly, this effect is exacerbated in a high host lipid environment when the *Tg*LIPIN is depleted (i.e., the more host lipid in the absence of TgLIPIN, more membrane, less TAG, high parasite death rate) the unlike wild-type parasites, which growth and survival is typically favored under higher host lipid/nutrient content [39]. This imbalance leads to an accumulation of free FAs, which are indicative and causative of lipotoxicity, and ultimately leads to the parasite’s death. It is interesting to note that gMVBs are highly formed under suppression of this protein in both low and high nutrient environment. The formation of gMVBs was previously observed to form solely under low host nutritional conditions, as seen by Amiar and colleagues (Fig 4). The parasite thus seems tricked into a false environment of low lipid levels, thinking that no lipids are available for the conversion of PA to DAG and for the formation of storage lipid droplets/TAGs. The parasite thus seems to overcompensate by continuing to scavenge host lipids that leads to an accumulation of PA, which unnaturally fuels PLs synthesis, then diverged into the endomembrane system of the parasite, affecting the membrane/structural integrity of the parasite.

Furthermore, phospholipases are suspected to play a crucial role in the recycling and reshuffling of lipids (Fig 4). Phospholipases are lipolytic enzymes that hydrolyze phospholipid substrates at specific ester bonds, releasing lysolipids and FA. *Tg*PL2 is one such phospholipase that localizes to the apicoplast and regulates PC and lysophosphatidylcholine (LPC) levels in the parasite [96]. This protein seems to be crucial in the lipid homeostasis of the apicoplast since its deletion leads to rapid organelle loss. Different LPLs can serve to generate FA that sources TAG and DAG synthesis, but their specific localization allows different function. *Pf*LPL1 is a dynamic protein of *P*. *falciparum*, which transits from parasite periphery/PVM into cytosolic vesicles that ends in a compartment closely localized to the food vacuole and rich in neutral lipids. *Pf*LPL1 generates FA that are used to synthesis neutral lipids, DAG, and TAG, which are used to promote hemozoin formation in the food vacuole, thus allowing essential heme detoxification by using host lipids as substrates. Depletion of *Pf*LPL1 is deleterious for the *Plasmodium* blood stage but can be rescued by addition of exogenous FA in the culture media, clue of the parasite plasticity. Closer to the host, *Pf*LPL3 localized to the intravacuolar TVN. *Pf*LPL3 allows to hydrolyze host phospholipids to generate FA, then used for DAG/TAG formation. These DAG/TAGs are mobilized during blood stages schizogony and thus serve as a tightly controlled reservoir of building blocks for bulk phospholipid synthesis in the parasite. Such process is like what was uncovered through the characterization of *Tg*LIPIN in *T*. *gondii* tachyzoites where host lipids are constantly scavenged, then stored for a timely use for membrane biogenesis specifically during asexual division.

## Physiological context

The ability of *T*. *gondii* to infect any nucleated cell resides in its strength/flexibility to metabolically adapt to their host (Fig 4). These intracellular parasites are in constant competition with their host for nutrient dependencies (not limited to just lipids, but includes other metabolites like glucose and glutamine), which is indirectly dependent on the environmental nutritional conditions [6,97,98]. Amiar and colleagues have also shown that *T*. *gondii* parasites under a low lipid environment up-regulates the FASII pathway [6]. Moreover, encountering different cell types in an organism has exposed the parasites to a wide panel of environments with different nutrient availability. Endemic areas affected by *Plasmodium*, in many cases, have a population largely affected by malnutrition. Parasites infect different cell types (hepatocytes, erythrocytes) and face certain changes in host nutrient; under amino acid starvation, parasite asexual replication within the red blood cells is reduced, and, instead, parasites enter a hibernating state [99]. Host LPC was revealed to be a critical nutrient that influences parasite growth and differentiation to the sexual stage of blood stages, with high serum levels of LPC responsible for the repression of sexual conversion [100].

Metabolic adaptation in a physiological context of these diseases caused by these parasites is becoming increasingly studied as it may holds the keys to how the parasite can sense and adapt to its host metabolic and nutritional conditions [35]. Phenotypic analyses recently indicated that only traits effected by extracellular host environment conditions evolved in *Toxoplasma* lab-adapted strains, indicating this importance in parasite propagation. Expression of genes involved in FASII pathway and ER FA elongation were up-regulated in extracellular parasites after lab adaptation.

Recent study uncovered the role of a *trans*-FA responsible for the host specificity of *Toxoplasma*. Data show that the accumulation of systemic linoleic acid (18:2) (25% to 46% in cat serum) due to the unusual absence of a Δ6 desaturase within feline intestines allows parasite oocyst propagation [101]. Such a metabolic lipid environment therefore provides a unique platform for the development of sexual stages otherwise absent in intermediate hosts, including humans. The fact that linoleic acid is toxic for *Toxoplasma* asexual stage tachyzoites also favors sexual stage development in the cat intestine environment. The enzyme Δ6 desaturase usually catalyzes the conversion of linoleic acid (C18:2) to arachidonic acid (C20:4), which, in turn, can participate in the formation of immune mediators like eicosanoids. Therefore, absence of this enzyme in feline intestines would also rescue the possibility of strong immune response toward parasite sexual stage.

In summary, Apicomplexa lipid metabolism is highly flexible and adapted under different host conditions. Giant strides have been taken in recent years to uncover the complex biology of this parasite and its intimate interactions with the human host. But major questions remain unanswered. Much of this is centered on the cryptic nature of host effectors, which are poorly conserved across eukaryotes and have coevolved specifically in *Toxoplasma* with the human host. The processes by which parasites scavenge FAs and potentially whole lipids are still in question. Similarly, the proteins responsible for sensing, and adapting to, low host nutritional content are still poorly defined. Recently, a novel kinase *Pf*KIN1 has been found to bear homology to the Snf1 protein found to regulate the nutritional response to starvation in yeast [102]. *Pf*KIN mutant parasites struggle to adapt to low nutritional environments in *P*. *berghei* parasites [102]. Future efforts will likely focus on the signaling network responsible for sensing and implementing this response. Furthermore, by establishing that host nutrient content determines the extent of parasite scavenging response and host remodeling, it will be well worth experimenting with different host cell types to establish which genes are essential under which conditions and/or cell types.

Future research on Apicomplexa metabolism will follow the current trend of the parasite’s adaptation in different host conditions. It will be important to elucidate this plasticity in different cell types, nutrient conditions, and how it may play a role in stage conversion. Collectively, the work described in this review and successive work is paving the road in identifying novel anti-Apicomplexa therapeutics, specifically concerning the metabolic battle between the host and parasite.
